# Decrease in Hemoglobin Levels during Acute Attacks in Patients with Idiopathic Recurrent Pericarditis: A Model of Anemia in Acute Disease

**DOI:** 10.3390/jcm13195944

**Published:** 2024-10-06

**Authors:** Francesca Casarin, Ruggiero Mascolo, Irene Motta, Maddalena Alessandra Wu, Emanuele Bizzi, Alice Pedroli, Giulia Dieguez, Giacomo Iacomelli, Lisa Serati, Lorena Duca, Silvia Maestroni, Enrico Tombetti, Maria Domenica Cappellini, Antonio Brucato

**Affiliations:** 1 Department of Internal Medicine, ASST Fatebenefratelli-Sacco, Fatebenefratelli Hospital, 20121 Milan, Italy; francesca.casarin@unimi.it (F.C.); emanuele.bizzi@asst-fbf-sacco.it (E.B.); alice.pedroli@unimi.it (A.P.); giulia.dieguez@unimi.it (G.D.); giacomo.iacomelli@unimi.it (G.I.); lisa.serati@unimi.it (L.S.); enrico.tombetti@unimi.it (E.T.); antonio.brucato@unimi.it (A.B.); 2 Department of Biomedical and Clinical Sciences, ASST Fatebenefratelli-Sacco, University of Milan, 20157 Milan, Italy; 3 Department of Clinical Sciences and Community Health, University of Milan, 20122 Milan, Italy; irene.motta@policlinico.mi.it; 4 SC Medicina ad Indirizzo Metabolico, Fondazione IRCCS Ca’ Granda Ospedale Maggiore Policlinico, 20122 Milan, Italy; lorena.duca@policlinico.mi.it (L.D.); maria.cappellini@unimi.it (M.D.C.); 5 Division of Internal Medicine, ASST Fatebenefratelli-Sacco, Luigi Sacco Hospital, University of Milan, 20157 Milan, Italy; maddalena.ale.wu@gmail.com; 6 Department of Internal Medicine, Ospedale Papa Giovanni XXIII, 24127 Bergamo, Italy; smaestroni@asst-pg23.it

**Keywords:** pericarditis, recurrent pericarditis, anemia, anemia of inflammation, inflammasome

## Abstract

**Background/Objectives:** Anemia during acute inflammation is not well described in the literature. We aimed to study whether patients develop a transient hemoglobin decrease during an acute attack of recurrent pericarditis. **Methods:** We retrospectively analyzed patients with recurrent pericarditis. The primary endpoint was the difference in hemoglobin levels during an acute attack and in the following remission. As secondary endpoints, we correlated this variation with laboratory and clinical features; we also evaluated the available baseline hemoglobin values. **Results:** Sixty-two patients, including thirty females (48.4%), with a median age of 39 years, were observed during an acute attack and remission. The attack indexed was the first in 21 patients and the second or the third in 41, with pre-attack hemoglobin levels available for the latter group. Median hemoglobin levels (IQR) were 13.8 (12.8–15.1) g/dL at baseline, 12.0 (11.2–13.4) during attacks and 13.6 (13.1–14.0) during remission (*p* < 0.001). The median hemoglobin reduction between an acute attack and remission was 1.4 g/dL. Their mean corpuscular volume remained in the normal range. Hb reduction significantly correlated with C-reactive protein (CRP) elevation, neutrophilia and the neutrophil-to-lymphocyte ratio, but not serosal involvement. Only CRP elevation remained associated with the variation of Hb in a multivariate analysis (*p* = 0.007). **Conclusions:** This study is a proof of concept: hemoglobin levels may decline rapidly during acute inflammation in correlation with CRP elevation, with transient normocytic anemia, followed by a rapid rebound. In this regard, idiopathic pericarditis may represent a pathogenetic model of this type of anemia.

## 1. Introduction

Anemia is defined by a hemoglobin concentration <12 g/dL for women and <13 g/dL for men. In chronically ill patients, anemia represents a common, well-studied condition, defined as the anemia of chronic diseases (ACD) or anemia of inflammation (AI) [1]. The prolonged and sustained activation of inflammation, as in infections, immune-mediated disorders, and solid and hematological malignancies, causes a reduction in hemoglobin. Chronic anemia can also occur in other conditions, such as chronic kidney disease, congestive heart failure, chronic lung disease, and obesity. ACD usually presents with mild normocytic anemia, reduced circulating iron concentrations, normal or reduced transferrin saturation, and normal or increased ferritin levels [1]. The most effective treatment for ACD is to treat the underlying condition, which can lead to the resolution of anemia. Three major pathophysiological pathways are involved in ACD/AI: iron depletion, erythropoiesis suppression, and, to a lesser extent, a reduced red blood cell lifespan [1].

Erythron, defined as the overall population of circulating red blood cells (RBCs) and their progenitors, depends on an equilibrium between erythropoiesis and the clearance of aged or damaged RBCs maintained by the reticuloendothelial system. This process is associated with a fine mechanism of iron recycling that involves the spleen, the liver, the bone marrow, the kidneys, and the duodenum.

Iron restriction is the most important pathophysiological mechanism of chronic anemia [2,3]: systemic inflammation induces the production of interleukin (IL)-6, produced by monocytes and macrophages, which inhibits transferrin production and triggers, through JAK2-STAT3 signaling, the hepatic production of hepcidin, an important acute-phase reactant [4]. Hepcidin binds the ferroportin, the only known transmembrane iron exporter, expressed in duodenal enterocytes, hepatocytes, and macrophages, causing its internalization and degradation. This results in reduced iron absorption in the duodenum and its sequestration in the reticuloendothelial system, with low circulating levels of ion [5,6,7]. Iron homeostasis plays an important role in regulating immune responses, sustaining the activity of leukocytes and contrasting infections [8,9]. Also, in COVID-19 infections, alterations in hepcidin-mediated iron metabolism were found but counterbalanced by the hypoxic stimulus [10,11]. Decreased erythropoiesis also contributes to chronic anemia through reduced erythropoietin (EPO) production and a diminished EPO-responsive cell pool in the bone marrow [12,13].

Moreover, the increased clearance of erythrocytes could also cause anemia during inflammation, a condition in which damage to RBCs and elevated phagocytic capacity could explain increased erythrocyte clearance [14]. Indeed, proinflammatory mediators (reactive oxygen species, NO, cytokines, complement mediators and antibodies [15]) could contribute to erythrocyte damage and destruction. Several mouse models showed that severe inflammation induces the activation of Interferon (IFN)-γ. This cytokine leads to recruitment from the circulation [16] and the activation of macrophages in the spleen and the liver (and, in some extreme cases, also in bone marrow), and depletes the CD47 on erythrocytes, enhancing their susceptibility to phagocytosis. These processes induce coagulopathy and erythrophagocytosis by hepatic and splenic macrophages, reducing erythroid cell survival [17,18].

On the other hand, the literature has not thoroughly described anemia related to acute inflammatory conditions [10,19]. In clinical practice, we have observed that hemoglobin levels significantly and rapidly decrease during an acute attack of recurrent pericarditis in some patients, which may even induce the physician to consider red cell transfusions. However, we observed that hemoglobin levels rapidly increase upon its resolution.

Our study aimed to investigate the variations in hemoglobin values seen during an acute attack of recurrent pericarditis, comparing them with those observed at baseline and during remission, while also evaluating the possible correlations with C-reactive protein (CRP) levels and other biomarkers, to elucidate a potential model of anemia during acute disease.

## 2. Materials and Methods

### 2.1. Patients

We analyzed retrospective data from two referral centers (Fatebenefratelli Hospital in Milan and Papa Giovanni XXIII Hospital in Bergamo, Italy) between January 2010 and May 2023. The data were collected from 62 consecutively enrolled patients who were diagnosed with recurrent pericarditis according to the 2015 European Society of Cardiology (ESC) guidelines [20]. These patients had complete serial blood cell counts. The local Ethics Committee approved the study (Comitato Etico di Area 1, Milan, Italy, Protocol number 2019/ST/222 on 23 July 2020). All patients provided informed consent.

Patients with cause-specific pericarditis (e.g., bacterial, neoplastic and autoimmune pericarditis) were excluded; patients with post-cardiac injury pericarditis were included. The study followed the STrengthening the Reporting of OBservational studies in Epidemiology (STROBE) reporting guidelines.

We collected demographic, clinical and laboratory data for each patient during an index attack. In our clinical practice, we observed that the severity of anemia seemed related to the severity of the inflammation, which was more intense in the first attacks. So, we aimed to assess the first attack, but the complete blood cell data set was available only in 21 patients during the first attack; the other 41 patients’ data were available during the second or the third attack. For these patients, pre-attack (baseline) hemoglobin values were also available.

To investigate the relationship between acute-phase reactants and iron metabolism, hepcidin, soluble transferrin receptor (sTfR) and Interleukin (IL)-6 were measured in two representative hospitalized patients during an acute attack and remission.

A baseline blood analysis and instrumental tests were conducted before administering specific therapies for the attack. A transthoracic echocardiogram was performed to determine the size of the pericardial effusion.

### 2.2. Outcomes

The primary outcome was the hemoglobin difference between an acute pericarditis attack and the following remission in all 62 patients. As secondary outcomes, we correlated this variation with inflammatory parameters and clinical features during the indexed attack; we also evaluated pre-attack (baseline) hemoglobin values, when available.

### 2.3. Statistical Analysis

Assuming a mean hemoglobin variation between the acute attack and remission of 1 g/dL and a 95% confidence interval width of at least 1 g/dL, a sample size of 45 patients allows us to obtain a Type 1 (alpha) error probability equal to 0.05 and a power of the study that is equal to 90%.

Continuous variables are described as median values and interquartile ranges (IQRs), whereas categorical variables are expressed as numbers and percentages (%). We used the Friedman test for paired data to define the hemoglobin difference between the baseline, indexed attack and remission. We used the Wilcoxon signed-rank test for paired data to define the mean corpuscular volume (MCV) difference between the indexed attack and remission. Spearman’s rho coefficient analyzed correlations of these variations with other laboratory parameters, while the Mann–Whitney U test assessed those with instrumental findings. We did not adjust the analysis of secondary outcomes for multiple comparisons.

In our multivariate regression model, we analyzed the relationship between changes in hemoglobin and other blood biochemistry parameters.

The statistical significance threshold was 0.05. Statistical analysis was performed using SPSS 28.0.1 (IBM, Armonk, NY, USA).

## 3. Results

### 3.1. Features of Study Cohort

Of the 62 enrolled patients, 30 (48.4%) were females. The median (IQR) age at the indexed attack was 39 (26 to 60) years, with most patients not being smokers (88.7%) and not having significant comorbidities (Table 1a). During the attack, pericardial effusion developed in 48 patients (77.4%), with a median (IQR) size of 6 mm (3.75 to 10.75) at ultrasonography; among them, cardiac tamponade occurred in 2 patients, and 4 patients needed pericardiocentesis. Pleural and peritoneal involvement was detected in 28 (45.2%) and 8 (12.9%) patients, respectively. The remission phase occurred after a median (IQR) period of 21 (16 to 25) days. Baseline hemoglobin values were available in 41 patients, tested between 1 and 3 months before the indexed attack (Table 1b).

Laboratory findings for the whole cohort showed a significant increase in CRP, white blood cells and neutrophil values during the acute attack, which markedly reduced in the remission phase, underlining the role of the inflammatory burst in acute pericarditis (Table 1c).

### 3.2. Hemoglobin Decreases during an Acute Attack

**Figure 1:**
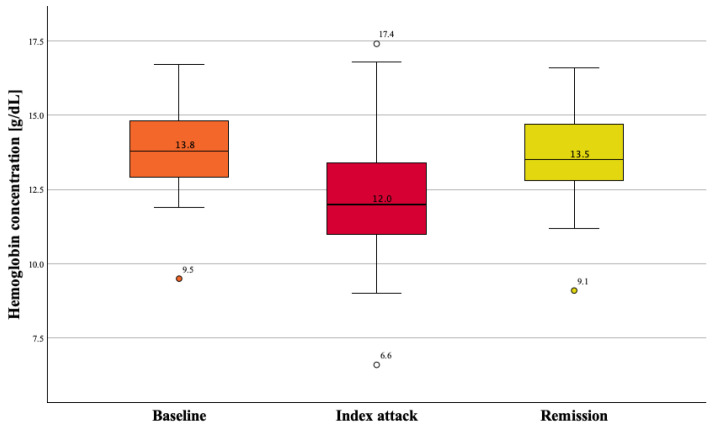
Box plot of paired hemoglobin values at baseline, during indexed attack and during remission in the 41 patients for which all data were available.

Hemoglobin levels during the indexed attack were significantly lower than those during remission [median (IQR): 12.0 (11.2 to 13.4) g/dL vs. 13.6 (13.1 to 14) g/dL], with a median reduction of 1.4 g/dL (*p* < 0.001), maintaining the medium corpuscular volume (MCV) within a normal range. We also recorded baseline pre-attack hemoglobin values in 41 patients: in these patients, their median (IQR) values of hemoglobin were 13.8 (12.8 to 15.1) g/dL at baseline, 12.0 (10.9 to 13.4) g/dL during the acute attack and 13.5 (12.7 to 14.9) g/dL during remission (*p* < 0.001 either for baseline vs. acute attack or for acute attack vs. remission) (Table 2a,b). Figure 1 shows the hemoglobin variation in patients with available pre-attack hemoglobin values.

Twenty-five patients (40.3%) who had anemia (defined as a hemoglobin concentration <12 g/dL for women and <13 g/dL for men) during their acute attack normalized their hemoglobin values in the remission stage. On the other hand, 24 patients (38.7%) had normal hemoglobin values during both the acute attack and remission and 11 (17.3%) remained anemic following the resolution of the acute attack (Table 3a,b).

### 3.3. Correlation between Hemoglobin and Other Laboratory and Clinical Parameters

This reduction in hemoglobin concentration was correlated with other inflammatory parameters, such as an increase in CRP values and leukocytes; this variation was mainly sustained by the rise of the neutrophil count, resulting in an elevated neutrophil-to-lymphocyte ratio (NLR) (Table 4). The decrease in hemoglobin values during the acute attack was not significantly correlated with the presence of pericardial, pleural and peritoneal effusions (Table 5).

In a multivariate analysis, only CRP elevation was confirmed to be associated with the variation of hemoglobin [R^2^ (es) = 0.212 (1.156), *p* = 0.005; β = 0.336, *p* = 0.007].

### 3.4. Anemia and Iron Metabolism in Two Representative Hospitalized Patients

Two male patients, aged 68 and 37 years, respectively, were admitted due to an attack of recurrent pericarditis.

During hospitalization, each patient had high levels of CRP associated with a rapid decrease in their hemoglobin concentration (Table 6a,b): starting from normal values of hemoglobin, they both rapidly reached values of as low as 10 g/dL in the absence of blood losses, other acute conditions or therapies that might contribute to such a reduction. Their mean corpuscular volume was normal. Once a proper treatment for pericarditis was established with anti-inflammatory drugs, the patients showed rapid clinical improvement, a reduction in neutrophilic leukocytosis and their CRP levels, and a slower rise in hemoglobin values without specific treatment for anemia, in the absence of other complications. Their blood iron concentration, transferrin levels and saturation were reduced during the acute attack, improving with the resolution of the inflammation.

At the onset of inflammation, elevated levels of IL-6 and hepcidin were found in both patients, which were associated with acute anemia and alterations of their iron homeostasis; as soon as their pericarditis improved, these two parameters markedly reduced, which was associated with improvements in their hemoglobin levels.

## 4. Discussion

In this study, we demonstrated for the first time that acute inflammation during a pericarditis attack is associated with transient normocytic anemia. Hemoglobin values dropped during the acute attack, while they were notably very similar before (baseline) and after (remission) the indexed attack (Figure 1). Indeed, our findings reveal that hemoglobin values decrease by approximately one and a half grams per deciliter during acute pericarditis compared to its quiescent phase. We also found that in acute pericarditis the decline of hemoglobin levels correlated with neutrophilia and elevated NLR and CRP values.

We have formally demonstrated this in the initial stages of pericarditis, when the inflammation is more intense. However, in our clinical experience, we have also observed variations in hemoglobin levels during subsequent recurrences, which are usually less pronounced. This is likely because the appropriate therapy was able to control the disease more rapidly.

In critical settings such as sepsis, major surgery, trauma and hemofiltration, anemia can develop abruptly through different mechanisms, even if blood losses, overwhelming infections and coagulopathies remain pivotal [21]. Boshuizen et al. identified rapid changes in iron homeostasis that were associated with elevated levels of IL-6 and ferritin [22]. Loftus et al. showed, in critical septic patients, that there is a correlation between reduced levels of hemoglobin and an increased concentration of proinflammatory cytokines [23]. Bateman et al. described persistent normochromic and normocytic anemia in ICU patients up to 6 months after their discharge, associated with a markedly reduced quality of life due to an inappropriate erythropoietin response and poor marrow red cell production [24]. Walsh et al. showed that 77% of survivors from the ICU remained anemic, with slow and incomplete recovery after their discharge [25].

The pathogenesis of idiopathic recurrent pericarditis is not well known, but the activation of the NLRP3-based inflammasome complex, responsible for the elevated production of IL-1β, seems to play a pivotal role [26]. This is also suggested by several studies showing the efficacy of treatment with anti-IL 1 agents [27,28,29].

The previously mentioned mechanisms could explain the development of acute anemia in many patients with acute pericarditis; inhibitory activity on hemopoietic stem cells and the hepcidin-mediated alteration of iron homeostasis could represent the principal mechanisms behind the acute reduction in hemoglobin.

Different studies have shown that IL-1β can hinder erythropoiesis and promote myelopoiesis by inhibiting GATA-1 and activating PU.1, two transcription factors crucial for the differentiation of hematopoietic stem cells [30,31]. Pre-clinical research in mouse models of a cryopyrin-associated periodic fever syndrome (CAPS) demonstrated correlations between the expression of NLRP3 and the reduction in erythrocytes and their progenitors [32].

Although our study does not aim to explain the cause of acute anemia during acute pericarditis, we observed lower serum iron and higher ferritin levels in the two representative hospitalized patients. These changes were associated with normal serum transferrin concentrations and its soluble receptor (sTfR), as seen in anemia in chronic diseases [1]. We also observed acutely increased concentrations of IL-6 and hepcidin during the acute attack in correlation with the high levels of CRP and severe anemia. Over time (days), we first observed an increase in CRP levels, followed by a progressive fall in hemoglobin; once the acute inflammation was solved, the CRP values progressively decreased, and, more slowly, the hemoglobin levels returned to the baseline.

Finally, in clinical practice, the abrupt reduction in hemoglobin that may be observed in some patients with acute recurrent pericarditis may influence their management and treatment, with there being a tendency to reduce ongoing therapy with non-steroidal anti-inflammatory drugs and corticosteroids mainly due to a suspicion of gastrointestinal bleeding. On the other hand, hemodilution induced by infusions does not explain the observed decrease in hemoglobin, since these patients were not treated with high doses of intravenous fluid, and most of them were febrile. Overall, we propose that an appropriate treatment of the inflammation can solve the attack while also reversing the decrease in hemoglobin levels.

### 4.1. Limitations

First, this is a retrospective study. Secondly, the sample size is small, but statistically adequate according to our sample size calculation; overall, recurrent pericarditis is a rare condition, and we enrolled patients from two tertiary centers in Italy for which serial reliable data were available. Moreover, comorbidities might influence hemoglobin fluctuations, but most of the patients in our study were under 40 years of age, non-smokers and there was a low incidence of significant comorbidities; thus, we are confident in having excluded the relevant comorbidities able to induce transient variations of hemoglobin. Finally, it is not possible to draw conclusions about iron homeostasis from just the two representative patients we studied in some detail.

### 4.2. Future Directions

This study firstly shows a new model of acute anemia related to acute inflammation; the pathogenesis of this fascinating condition requires further studies aiming to reveal the involved molecular pathways and the interplays between the possible main players, particularly iron, hepcidin, sTfR, IL-6 and IL-1.

## 5. Conclusions

Our study showed that hemoglobin values decrease acutely during an acute attack of pericarditis, which could represent an intriguing model of anemia in acute disease.

The appropriate treatment of inflammation can solve the attack while also reversing the decrease in hemoglobin levels. Its pathogenetic mechanism could be linked to inflammasome activation and the production of IL-1 and IL-6, which lead to an increase in hepcidin and the inhibition of erythropoiesis. Of course, further studies are needed to define how acute inflammatory pathways may acutely influence hemopoiesis.

## Figures and Tables

**Table 1:** Demographic, clinical and laboratory findings of the study cohort (N = 62).

(a) Demographic Characteristics *	
Age at index attack, years—median (IQR)	39 (26–60)
• <40 years	32 (51.6%)
• 40–65 years	14 (22.6%)
• >65 years	16 (25.8%)
Gender F:M	30 (48.4%): 32 (51.6%)
Smoking habits	
• Former	20 (32.3%)
• Active	7 (11.3%)
• Non-smoker	35 (56.4%)
Comorbidities	
• Arterial hypertension	12 (19.4%)
• Asthma	4 (6.5%)
• Type 2 diabetes mellitus	1 (1.6%)
• Thyroid dysfunction	3 (4.8%)
• Atrial fibrillation	2 (3.2%)
(b) Clinical Characteristics at Indexed Attack *	
Pericardial effusion	48 (77.4%)
Size of pericardial effusion [mm]—median (IQR)	6 (3.75–10.75)
Cardiac tamponade	2 (3.2%)
Pericardiocentesis	4 (6.5%)
Pleural effusion	28 (45.2%)
Ascites	8 (12.9%)
Therapy	
• Nonsteroidal anti-inflammatory drugs	55 (88.7%)
• Colchicine	37 (59.7%)
• Corticosteroids	22 (35.5%)
• Anakinra	3 (4.8%)
Remission time, days—median (IQR)	21 (16–25)
(c) Laboratory Findings **	
Maximum C-reactive protein, CRP [mg/L]	
• Indexed attack	70 (29–197.25)
• Remission	5 (2–5)
• Difference (Delta CRP)	68 (24–174.75)
White blood cells count, WBC [cells/µL]	
• Indexed attack	11,400 (6660–13,225)
• Remission	7170 (6275–8030)
• Difference (Delta leukocytes)	4359 (1673–6187)
Absolute neutrophil count, ANC [cells/µL]	
• Indexed attack	8694 (5826–10,708)
• Remission	4150 (3616–5993)
• Difference (Delta neutrophils)	4390 (1410–6717)
Absolute lymphocyte count, ALC [cells/µL]	1640 (1210–3067)
Neutrophil–lymphocyte ratio, NLR	5.93 (3.27–7.41)

IQR: interquartile range. * Unless otherwise specified, expressed as count (percentage %). ** Expressed as median (IQR).

**Table 2:** Variations in hemoglobin levels and mean corpuscular volume (MCV) at baseline, during the indexed attack and at remission.

**(a) Variations in hemoglobin levels**		
Hemoglobin Levels *	Hb [g/dL]	*p*-Value **
Baseline (N = 41)	13.8 (12.8 to 15.1)	
Indexed attack (N = 62)	12.0 (11.2 to 13.4)	
Remission (N = 62)	13.7 (13.1 to 14.0)	
Baseline–Indexed attack	1.5 (1.1 to 2.3)	<0.001
Remission–Indexed attack	1.4 (0.9 to 1.98)	<0.001
Baseline–Remission	0.2 (0.1 to 0.3)	0.045
**(b) Variations in MCV**		
Mean Corpuscular Volume *	MCV [fL]	*p*-Value **
Indexed attack (N = 62)	85 (83 to 86.7)	
Remission (N = 62)	85 (83 to 88)	
Difference (Delta MCV)	0.5 (−1.0 to 2.75)	0.083

(**a**) * Expressed as median (IQR). ** Friedman test for paired data, *p* < 0.05, with Bonferroni’s correction. (**b**) * Expressed as median (IQR). ** Wilcoxon signed-rank test for paired data, *p* < 0.05.

**Table 3:** Presence of anemia * during acute attack compared to that at baseline and remission.

**(a) Presence of Anemia during Indexed Attack and at Remission**			
	**No Anemia at Remission**	**Anemia at Remission**	**Total**
No anemia during attack	24 (38.7%)	2 (3.2%)	26 (41.9%)
Anemia during attack	25 (40.8%)	11 (17.3%)	36 (58.1%)
Total	49 (79.5%)	13 (20.5%)	62
**(b) Presence of Anemia at Baseline and during Indexed Attack**			
	**No Anemia at Remission**	**Anemia at Remission**	**Total**
No anemia during attack	16 (39.1%)	1 (2.4%)	17 (41.5%)
Anemia during attack	14 (34.1%)	10 (24.4%)	24 (58.5%)
Total	30 (73.2%)	11 (26.8%)	41

* Anemia: hemoglobin concentration <12 g/dL for women and <13 g/dL for men.

**Table 4:** Correlation of variations in hemoglobin levels (between remission and indexed attack) with other laboratory variables.

**Correlations of Delta Hb with:**	***ρ*** **(95%CI)**	***p*** -Value *
Age at event	0.005 (−0.253 to 0.261)	0.972
Delta CRP	0.391 (0.149 to 0.589)	0.002
Delta leukocytes	0.304 (0.051 to 0.520)	0.016
Delta neutrophils	0.426 (0.190 to 0.615)	<0.001
Maximum CRP at indexed attack	0.387 (0.145 to 0.586)	0.002
Maximum white blood cells count at indexed attack	0.279 (0.023 to 0.500)	0.028
Maximum neutrophil count at indexed attack	0.374 (0.130 to 0.576)	0.003
Maximum lymphocyte count at indexed attack	−0.064 (−0.315 to 0.196)	0.624
NLR at indexed attack	0.278 (0.022 to 0.499)	0.029
Size of pericardial effusion at indexed attack	0.108 (−0.153 to 0.355)	0.404

95%CI: 95% confidence interval; CRP: C-reactive protein; NLR: neutrophil-to-lymphocyte ratio. * Spearman’s rho coefficient, *p* < 0.05.

**Table 5:** Correlation between variations in hemoglobin levels (between remission and indexed attack) and clinical features of pericarditis.

Clinical Features *	**Delta Hb in Presence [g/dL]**	**Delta Hb in Absence [g/dL]**	***p*** -Value **
Pericardial effusion	1.38 (0.97 to 2.06)	1.75 (0.45 to 1.94)	0.846
Pleural effusion	1.68 (1.02 to 2.56)	1.35 (0.90 to 1.80)	0.145
Ascites	1.65 (0.77 to 2.39)	1.40 (0.94 to 1.90)	0.629

* Expressed as median (IQR). ** Mann–Whitney U test, *p* < 0.05.

**Table 6:** Laboratory findings before, during and after hospitalization of two representative patients.

**(a) 68-Year-Old Male Patient**						
	**Baseline (36 Days before Admission)**	**Day 0 (Emergency Room for Acute Attack)**	**Day 1 after Admission**	**Day 2 after Admission**	**At Discharge (Day 4 after Admission)**	**30 Days after Discharge**
Hb [g/dL]	15.0	12.6	10.2	9.4	10.8	12.0
Hct [%]	47	38	30	28	32	35
MCV [fL]	85.8	84.6	83.4	84.3	84.3	84.6
CRP [mg/L]		223.6	225.5	105.9	61.6	0.3
WBC [/µL]	7410	11,390	5490	5150	4980	5380
Neutrophil count [/µL]	3490	9112	4392	3657	3020	3570
Lymphocyte count [/µL]	3230	2278	1098	1494	1500	1300
Serum iron [μg/dL]					46	
Ferritin [μg/L]					572	
Transferrin [g/L]					1.7	
Transferrin saturation [%]					19	
Vitamin B12 [ng/L] (n.v. > 300)					161	
Folate [μg/L] (n.v. > 4)					4.7	
Haptoglobin [g/L] (n.v.: 0.3–2)					1.9	
LDH [UI/L]					116	
Hepcidin [ng/mL] (n.v. < 21.8)				178		45
sTfR [mg/L] (n.v. 1.8–4.6)				3.27		2.66
IL-6 [pg/mL] (n.v. 0–10)				110		25.3
**(b) 37-Year-Old Male Patient**						
	**Baseline (120 Days before Admission)**	**Day 0 (Emergency Room for Acute Attack)**	**Day 1 after Admission**	**Day 3 after Admission**	**At Discharge (Day 4 after Admission)**	**30 Days after Discharge**
Hb [g/dL]	15.0	12.0	10.2	11.2	12.8	13.8
Hct [%]	47	36	31	34	39	43
MCV [fL]	91.8	88.0	86.4	89.8	86.8	90.3
CRP [mg/L]		251.3	252.5	105.7	59.4	4.6
WBC [/µL]	7410	14,670	10,670	6910	7030	10,210
Neutrophil count [/µL]	3490		7390	3000	4320	4520
Lymphocyte count [/µL]	3230		2030	3170	1880	4720
Serum iron [μg/dL]			13			76
Ferritin [μg/L]			743			402
Transferrin [g/L]			1.54			2.67
Transferrin saturation [%]			6			20
Vitamin B12 [ng/L] (n.v. > 300)			380			
Folate [μg/L] (n.v. > 4)			4.1			
Haptoglobin [g/L] (n.v.: 0.3–2)						0.04
LDH [UI/L]						178
Hepcidin [ng/mL] (n.v. < 21.8)				63		48
sTfR [mg/L] (n.v. 1.8–4.6)				3.04		2.66
IL-6 [pg/mL] (n.v. 0–10)				67.3		23.8

Hb: hemoglobin; Hct: hematocrit; MCV: mean corpuscular volume; CRP: C-reactive protein; WBC: white blood cells; LDH: lactate dehydrogenase; sTfR: soluble Transferrin Receptor; IL-6: Interleukin-6.

## Data Availability

The data supporting this study’s findings are available on request from the corresponding author. The data are not publicly available due to privacy or ethical restrictions.
